# Identification of genetic variants of *LGI1* and *RTN4R* (NgR1) linked to schizophrenia that are defective in NgR1–LGI1 signaling

**DOI:** 10.1002/mgg3.215

**Published:** 2016-03-11

**Authors:** Rhalena A. Thomas, Amirthagowri Ambalavanan, Guy A. Rouleau, Philip A. Barker

**Affiliations:** ^1^ Department of Neurology and NeurosurgeryMontreal Neurological InstituteMcGill University3801 UniversityMontrealQuebecH3A 2B4Canada; ^2^ Department of Human GeneticsMcGill University1205 Dr Penfield AvenueMontrealQuebecH3A 1B1Canada; ^3^ Department of BiologyUniversity of British ColumbiaKelownaBC. V1V 1V7Canada

**Keywords:** LGI1, NgR1, RhoA, RTN4R, schizophrenia, synapse formation

## Abstract

**Background:**

The protein NgR1 is encoded by *RTN4R*, a gene linked to schizophrenia. We previously reported NgR1 as receptor for the epilepsy‐linked protein LGI1. NgR1 regulates synapse number and synaptic plasticity, whereas LGI1 antagonizes NgR1 signaling and promotes synapse formation. Impairments in synapse formation are common in neurological disease and we hypothesized that an LGI1–NgR1 signaling pathway may contribute to the development of schizophrenia.

**Methods:**

We screened two unrelated schizophrenic populations for variants in *RTN4R* and *LGI1* using whole exome sequencing and Sanger sequencing. We tested the ability of LGI1 to bind rare coding variants of NgR1 using a cell surface binding assays and the signaling ability of NgR1 using COS7 cell‐spreading assays.

**Results:**

We observed a previously reported rare coding variant in *RTN4R* (c.1195C>T, pR399W). We report the first *LGI1* mutations to be identified in individuals with schizophrenia. Three different *LGI1* mutations were found, two missense mutations (c.205G>A, p.V69I) and (c.313G>A, V105M), and an intronic variant (g.897T>C) that likely leads to a protein truncation. We found NgR1^R119W^ and NgR1^277C^ have a reduced ability to bind LGI1 in a cell surface binding assay. COS7 cell‐spreading assays reveal that NgR1 mutants are impaired in their ability to mediate RhoA activation.

**Conclusion:**

Variants in NgR1 and LGI1 may be associated with schizophrenia and variants in NgR1 found in schizophrenic patients have impaired LGI1–NgR1 signaling. Impaired LGI1–NgR1 signaling may contribute to disease progression.

## Introduction

Schizophrenia (SCZ) is genetically heterogeneous and several genes have been reported to be associated with its development and progression. A recent large‐scale genome‐wide association study (GWAS) identified 108 common variants associated with schizophrenia (Schizophrenia Working Group of the Psychiatric Genomics Consortium 2014), with many loci present within noncoding regions. The genetic link to SCZ is well established but these common variants only account for a 10% of the risk of disease (Gibson 2011). SCZ may be caused by many different rare coding variants, possibly with a different set of mutations in each patient. Changes in synaptic pruning, synaptic plasticity, memory, and myelination have all been linked to SCZ (Hacohen et al. 2014; Maćkowiak et al. 2014; Murray et al. 2014). NgR1, encoded by *RTN4R*, has roles in all of these processes and interestingly, *RTN4R* has a genetic link to schizophrenia (OMIM # 605566). NgR1 was initially identified as a receptor for myelin‐associated growth inhibitors (MAIs): myelin‐associated glycoprotein (MAG), oligodendrocyte glycoprotein (OMGP), and NogoA (reticulon 4 (RTN4)) (Fournier et al. 2001; Liu et al. 2002a; Wang et al. 2002b). Subsequently NgR1 was shown to have a role in restricting plasticity (McGee et al. 2005) and to be required for long‐term depression (Lee et al. 2008). Most recently, NgR1 has been shown to limit synapse number and regulate addition and removal of dendritic spines (Wills et al. 2012; Akbik et al. 2013).

NgR1 function not only overlaps with processes involved in SCZ, there is also genetic evidence linking NgR1 to the disease. NgR1 is located at 22q11, deleted in a subtype of SCZ (Liu et al. 2002b; Perlstein et al. 2014). The 22q11 deletion confers an 80‐fold increase in risk of schizophrenia (Baron 2001). Association studies provide support for a link between SCZ and NgR1 in Italian, Caucasian American, and South African populations (Sinibaldi et al. 2004; Hsu et al. 2007; Budel et al. 2008), however there is no association in several Chinese and Japanese populations (Hsu et al. 2007; Meng et al. 2007; Budel et al. 2008; Jitoku et al. 2011). Intriguingly, several rare coding variants in *RTN4R* have been uncovered in SCZ populations (Sinibaldi et al. 2004; Hsu et al. 2007; Budel et al. 2008). Budel et al. (2008) reported functional impairments in NgR1 ligand binding and neurite outgrowth inhibition in several human *RTN4R* rare coding mutations. NgR1 null mice have a delay in learning spatial memory tasks (Budel et al. 2008) and consolidation of fear extinction (Park et al. 2014). Mice constitutively expressing NgR1 from a CamKII promoter no longer downregulate NgR1 in response to activity. In the Morris water maze reference memory task, NgR1 overexpressing mice have impaired performance 40 days after training (Karlén et al. 2009).

We previously identified LGI1 as a novel ligand for NgR1 that acts antagonistically to block the action of MAIs (Thomas et al. 2010). A clear role for LGI1 in circuitry formation and synaptic transmission in humans has been shown by two disease states (OMIM 604619). Mutations in LGI1 cause autosomal dominant lateral temporal lobe epilepsy (ADLTLE) (Morante‐Redolat et al. 2002) and antibodies directed against LGI1 are found in one form of autoimmune limbic encephalitis (LE) (Lai et al. 2010). LE caused by LGI1 antibodies is characterized by sudden confusion, memory loss, psychosis, and seizures (Lai et al. 2010). Deletion of LGI1 in mice results in early postnatal spontaneous seizures followed by death (Chabrol et al. 2010; Fukata et al. 2010; Yu et al. 2010). Additionally, the gene location of *LGI1* is at a site linked to SCZ susceptibility (Fallin et al. 2003; Lerer et al. 2003). We have previously shown LGI1 permits neurite outgrowth on myelin substrates and prevent rat dorsal root ganglia (DRG) growth cone collapse induced by myelin, processes mediated by RhoA activation (Thomas et al. 2010).

In this study, we analyzed two unrelated schizophrenia populations for mutations in *LGI1* and *RTN4R* (NgR1). We searched whole exome sequencing data from 35 schizophrenia trios (parents and child groups) samples recruited for previous studies (Girard et al. 2011; Ambalavanan et al. 2015). Childhood onset schizophrenia (COS) is a rare disorder where children over the age of 7 begin to experience schizophrenic symptoms. To date no investigation into variations in *RTN4R* have been performed in a COS population but intriguingly, 6% of COS patients carry the 22q11 deletion.

We analyzed 20 whole exome sequences from patients affected with COS and 15 other trios that were affected by schizophrenia for variants in *RTN4R* and *LGI1*. Additionally, we screened 493 unrelated individuals from SCZ patient samples covering all coding regions and splice site junctions of *RTN4R* and *LGI1*. We identified one rare coding variant in *RTN4R* within the COS population, a mutation previously identified in SCZ. Furthermore, we uncovered two coding variants in *LGI1* and two intronic variants in *LGI1* within the 493 patient samples. This is the first report of variants in *LGI1* associated with schizophrenia. We next tested the functional effects of rare coding variants in the gene encoding NgR1 by producing mutant forms of NgR1 protein encoded by the amino acid substitutions. We found reduced binding of two mutant NgR1 proteins and impaired functions in a COS7 cell‐spreading assay. The balance between LGI1 and NgR1 activation of RhoA at synapses regulates developmental synapse number, suggesting that SCZ mutations in NgR1 may lead to dysregulation of synapse number and disease.

## Materials and Methods

### Ethical compliance

Our study uses previously published DNA samples and patient clinical data that were collected in accordance with French ethics committees (Girard et al. 2011) and the McGill University Institutional Review Board (A12‐M69‐98).

### Whole exome sequencing

In this study, we analyzed 35 schizophrenia samples from our whole exome sequencing in‐house data. These samples were recruited as part of our previous studies (Girard et al. 2011; Ambalavanan et al. 2015). Among those 35 samples, 20 from patients affected with childhood onset schizophrenia (COS) (Awadalla et al. 2010; Piton et al. 2011) and 15 other trios were affected by schizophrenia (Girard et al. 2011). We screened for variants in our candidate genes, *RTN4R* and *LGI1*. The reference sequences in GenBank are KR709468.1 (*LGI1*) and KR710415.1 (*RTN4R*). There is an average of 88% coverage at 10× in the candidate genes of COS and 70% covered at 5× in SCZ. The capture kits used for COS samples were SureSelect^XT^ Human All Exon V4 kit (Agilent Technologies Inc., Mississauga, ON, Canada) and for the SCZ samples were SureSelect Human All Exome Kit V1 and the captured libraries were sequenced in Illumina HiSeq2000 and GATIIX platform at the McGill University and Génome Québec Innovation Centre (Montréal, Canada) (Girard et al. 2011; Ambalavanan et al. 2015). The average coverage of *RTN4R* is 176 bases in SCZ and 2706 bases in COS, and the average coverage of *LGI1* is 2330 bases in SCZ and 5627 bases in COS (coverage is calculated for all the exons with 6 bp flanking region in the intron–exon border). The exome coverage is lower than current standards as these samples were collected analyzed in 2011; many recent improvements have been made in sequence and capture systems.

### Sanger sequencing

A genetic screening panel composed of 493 additional schizophrenia patients, without exome data, was selected from the unrelated individuals from patient samples of European Caucasian ancestry used in a previous study (Piton et al. 2011). All coding regions and splice site junctions of *RTN4R* and *LGI1* were amplified and sequenced using Sanger sequencing method. The designed primer sequences are provided in the Table 3. PCR products were sequenced at the McGill University and Génome Québec Innovation Centre (Montréal, Canada) and the sequences were analyzed with Mutation Surveyor v.4.0 (SoftGenetics, State College, PA). Primer sequences are shown in Table 1.

**Table 1:** Primer sequences for *LGI1* and *RTN4R* used for Sanger sequencing

LGI1_ex1_F	CCAGAAGCCCTGTTCATGGT
LGI1_ex1_R	CATGCAAAGCCCCAAATCCA
LGI1_ex2_F	GAGAAACCTGTAGCCGATTCA
LGI1_ex2_R	CGCAAACAAACCCATCTACC
LGI1_ex3‐4_F	TGAGAGATAAAAGCAGCCAAGA
LGI1_ex3‐4_R	GGTGCATTAACCACAGGTGA
LGI1_ex5_F	TGGGTGTTGAAGTGAACAGG
LGI1_ex5_R	CACCCCGTCAAAGTCCTTTAT
LGI1_ex6_F	CGGGTAAGGTCATTCTGCAC
LGI1_ex6_R	GCTAATACCTCTTTCCTTGGCTA
LGI1_ex7_F	CCTCGAAGGATTTTGATTGC
LGI1_ex7_R	AAGCATTCCCCTATACCACTCA
LGI1_ex8.1_F	GCTGATTTGGGTGGAAGTTG
LGI1_ex8.1_R	GCTTCACTGCGTACACATCC
LGI1_ex8.2_F	AGTAGTTCCCAGCGTCCTGT
LGI1_ex8.2_R	CATCATGCATTGAGTTCATCC
RTN4R_ex2_1_F	CAGCTTCTCCAGTACCCCTG
RTN4R_ex2_1_R	TGCAGGAAGAGGTGTGTGAG
RTN4R_ex2_2_F	TACACACGCTGCACCTGG
RTN4R_ex2_2_R	GCTCCAGTACTGAGGCCTTG
RTN4R_ex2_3_F	CTAGCTGCCAATGACCTGC
RTN4R_ex2_3_R	CGTGGAGAGAGACCCCG

### Statistical and bioinformatics analysis

Rare variants with less than 1% minor allele frequency were identified in the affected patients. For variants reported in Exome Variant Server (EVS) (NHLBI GO Exome Sequencing Project (ESP), Seattle, WA (http://evs.gs.washington.edu/EVS/), the allelic frequency of variants identified from our cohort were compared with the EVS using Fisher's exact test. For analysis of possible effects of missense variants in all genes, we used web based on the predictions of online prediction tools such as PolyPhen‐2 (Polymorphism Phenotyping‐2) (http://genetics.bwh.harvard.edu/pph2/), SIFT (Sorting Intolerant From Tolerant) (http://sift.bii.a-star.edu.sg/), and Mutation Taster (http://www.mutationtaster.org/). To test the pathogenic potential of our variants, we have used Residual Variant Intolerant Score (RVIS). This genome‐wide scoring system assesses the functional variation of human genes based on the single nucleotide variants in EVS. The RVIS percentile gives an indication as to whether a gene is “tolerant” or not to changes. The RVIS is calculated software (http://chgv.org/GenicIntolerance/).

### AP‐binding assays

Qualitative and quantitative AP‐binding assays were performed as previously described (Thomas et al. 2010). For the quantitative AP‐binding assay, COS7 cells were transfected with various constructs using Lipofectamine 2000 (Invitrogen, Waltham, MA USA). Live cells were incubated with 5 nmol/L AP‐LGI1 for 1.5 h prior to extensive washes. Bound AP‐LGI1 was quantified by OD_405_ to visualize PNPP substrate. To compare AP‐LGI1 binding between conditions, background AP‐LGI1 binding to mock was subtracted from all other conditions. The results were then normalized to the relative levels of NgR1 and mutant NgR1 expression determined by an adapted ELISA. Cells expressing various NgR1 plasmids were split in 96‐well plates one set for AP‐binding assays and one for the ELISA assays. Cells were fixed in PBS plus 4% paraformaldehyde and 4% sucrose. Next cells were incubated with blocking solution PBS plus 3% BSA, then incubated in anti‐NgR1 (R&D AF1440) 1/2000 in blocking solution. The NgR1 antibody recognizes all NgR1 constructs used in these experiments. Cells were washed, returned to blocking solution, and then incubated with secondary antibody conjugated to horseradish peroxidase. Levels of NgR1 were quantified with ABTS (3‐ethylbenzthiazoline‐6‐sulfonic acid, Sigma Cat. No. A‐1888), 0.1 mol/L citric acid, adjust pH to 4.35 with NaOH.

### COS7 cell‐spreading assay

COS7 cell‐spreading assay was performed as previously described by Zeinieh et al. (2014). Briefly, COS7 cells were maintained in DMEM with 10% bovine calf serum, 2 mmol/L l‐glutamine, and 100 *μ*g/mL penicillin/streptomycin, and transfected using lipofectamine 2000 (Invitrogen). Cells were trypsinized and reseeded at low cell density on glass coverslips (12 mm; Fisher Scientific, Ottawa, Ontario, Canada), coated with laminin (0.5 *μ*g/mL) 48 h post transfection. Cells were fixed in 4% PFA in PBS for 30 min at room temperature, 24 h after seeding. Coverslips were washed with PBS and incubated with rhodamine‐tagged WGA (5 *μ*g/mL) in PBS for 10 min at room temperature, then washed with PBS (3 × 5 min) and then quickly with water before mounting with antifading mounting media (Dako). Imaging was performed using a 40× objective on a Zeiss Axioskop fluorescent inverted microscope equipped with Xenon illumination, and images were captured using Zen software (Zeiss, North York, Ontario, Canada). The area of each cell determined using the measure tool in image J.

## Results

### Identification of rare variants in *RTN4* and *LGI1* genes associated with schizophrenia

**Figure 1:**
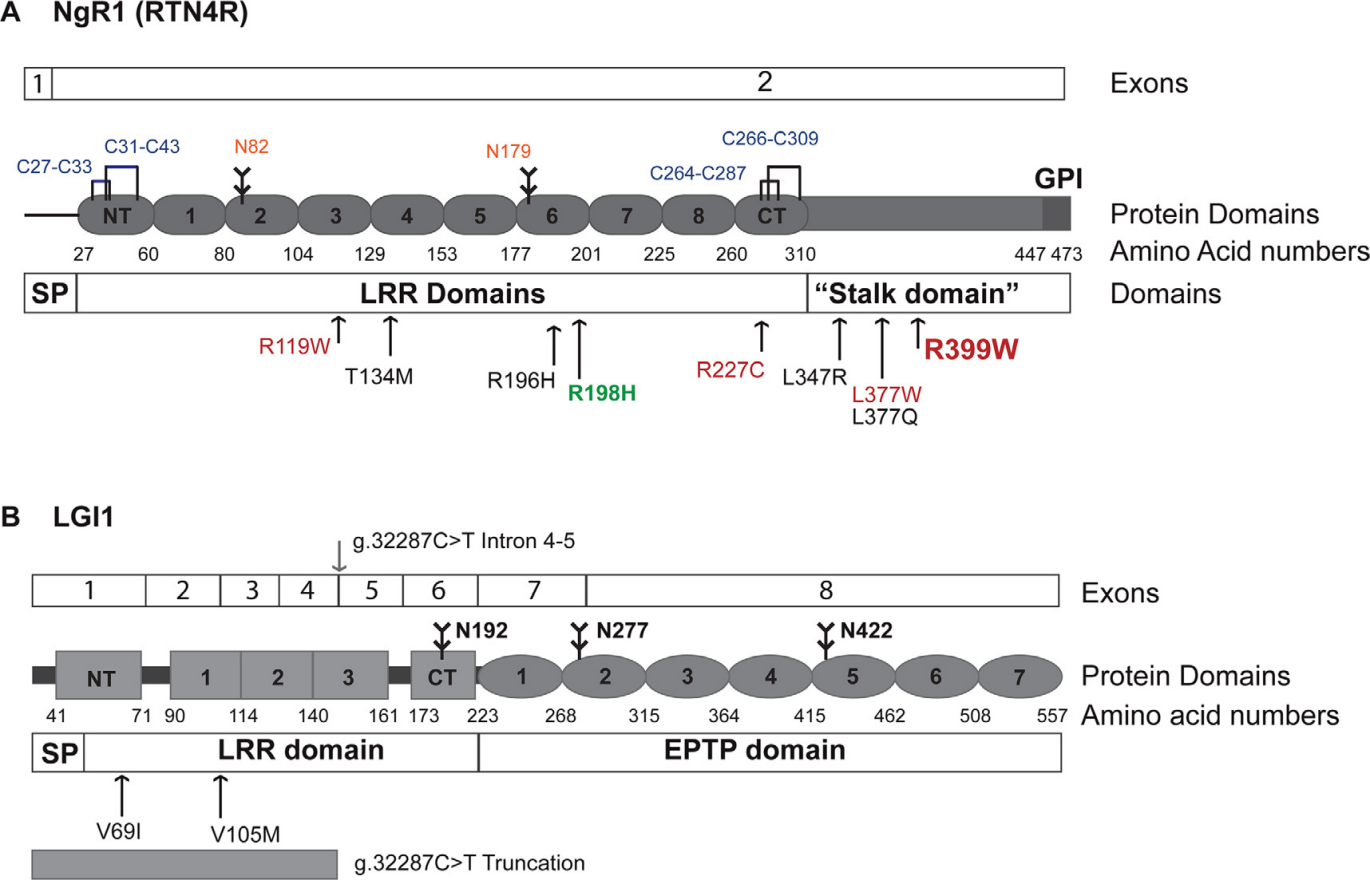
Schematics of NgR1 and LGI1 protein structure and exons. (A) Structure of NgR1 protein and exons from *RTN4R* are indicated. The amino acid numbers corresponding to protein domains are written below the schematic. Glycosylation sites are indicated by branched lines and amino acid residues in orange, the disulfide bonds are indicated by bridges and amino acids numbers are indicated in blue. All genetic changes are indicated with arrows. Previously published coding variants associated with schizophrenia (SCZ) are indicated and variants predicted to be deleterious are indicated in red. The mutation R399W identified in a COS patient in this study is indicated in larger bold font. The R189H mutant expressed in a transgenic mouse is indicated in green (B) Structure of LGI1 protein with exons indicated. Glycosylation sites are indicated by branched lines. Coding variants identified in SCZ patients are indicated with arrows. The intronic splice variant is indicated at the site between the exons 4 and 5 at the top of the schematic. The resulting truncation protein that could occur from the splice site variant is indicated at the bottom of the schematic.

The presence of novel nonsynonymous variants in *RTN4R* was previously reported in schizophrenia. Interestingly, here we identified one of the previously reported nonsynonymous *RTN4R* variants (R399W) within our COS trios (Table 2). The R399W variant was inherited from the patient's father, who does not suffer from schizophrenia. This indicates the variant is not likely disease causing or has low penetrance. However, in silico analyses using SIFT and Polyphen‐2 and MutationTaster31 each indicated a deleterious change in protein sequence (Table 3). The residue R399W is indicated on the schematic of NgR1 shown in Figure 1 A. NgR1 contains an N‐terminal (NT) leucine‐rich repeat (LRR) domain, eight LRR domains, CT‐LRR domain, a stalk domain, and a GPI anchorage site. The LRR domains form a curved banana structure and contain ligand‐binding regions for NogoA, MAG, OMGP, and LGI1 (He et al. 2003). The stalk region is the interaction site for coreceptor p75NTR or TROY and is needed for RhoA activation (Wang et al. 2002a).

**Table 2:** Variants identified in *RTN4R* and *LGI1* and their occurrence in schizophrenia (SCZ) and COS cohort

Gene	Genomic position	Nucleotide variant	dbSNP	EVS minor allele frequency
RTN4R	chr22:20,229,461	NM_023004.5:c.1195G>A	rs200119628	NA
LGI1	chr10:95,518,106	NM_005097.2:c.205G>A	rs147469708	0.034
LGI1	chr10:95,537,161	NC_000010.10:g95537161G>A	novel	NA
LGI1	chr10:95,518,462	NC_000010.10:g95518462T>C	rs143132529	1.0063

*RTN4R* variant identified in COS cohort and *LGI1* variants identified in European SCZ population are listed. Genomic position, nucleotide variants are indicated. dbSNP lists the previously published SNPs. NA indicates no alleles are listed in the Exome Variant Server (EVS) server. GenBank reference sequences *LGI1* (KR709468.1) and *RTN4R* (KR710415.1).

**Table 3:** In silico analysis of mutations found in *RTN4R* and *LGI1* in schizophrenia (SCZ) and COS cohorts

Gene	Nucleotide variant	AA	Location	SIFT	PolyPhen	Mutation Taster31	RVIS (%)
RTN4R	chr22:20,229,461	R399W	Stalk	Damaging	Probably damaging	Disease causing	7.05
LGI1	chr10:95,518,106	V69I	NT‐LRR	Tolerated	Benign	Disease causing	14.4
LGI1	chr10:95,537,161	V105M	LRR1	Tolerated	Benign	Disease causing	
LGI1	chr10:95,518,462	NA	Intron 4‐5	NA	NA	Disease causing	

The nucleotide variants resulting in coding mutation are indicated. Where the nucleotide change is located in an intronic region and there is no amino acid change, this is indicated by NA (not applicable). Location indicates the site within the exon protein‐coding region or which intron contains the variation when intronic. Results of analysis from three different programs used to predict the effect of a coding variant are listed. SIFT and PolyPhen do not analyze intronic variants. GenBank reference sequences *LGI1* (KR709468.1) and *RTN4R* (KR710415.1).

LRR, leucine‐rich repeat; SIFT, sorting intolerant from tolerant.

We have previously reported that LGI1 is a specific ligand for NgR1 and that LGI1 and Nogo‐66 compete for an overlapping binding site on NgR1 (Thomas et al. 2010). The LGI1 protein contains a NT‐LRR, 3 LRR, and CT‐LRR domain, and 8 ETPT domains (Morante‐Redolat et al. 2002). Both domains are involved in protein–protein interactions and the EPTP domain is needed for binding to another LGI1 receptor, ADAM22 (Fukata et al. 2006). In this current study, we identified three different variants in *LGI1* from the sequencing data, listed in Table 2.

We observed two missense variants and one mutation at intron/exon splice site. Of the *LGI1* variants identified, the missense V69I does not appear to be significantly associated with the disease; a *P* value of 0.56 (Fisher's exact test) can be calculated by comparing the frequency of this allele between cases and control individuals from the exome variant server (EVS). However, rare variants present less frequently in EVS database than in the disease cohort suggest that there could be rare LGI1 variants in subjects with psychiatric diseases. MutationTaster31 predicts that LGI1 coding mutations V69I and V105M are likely disease causing (Table 3). However, SIFT and PolyPhen predict these changes are not likely to be harmful. The other variant identified in *LGI1* is located at an intronic splicing site. According to MutationTaster31, the *LGI1* intronic variant g.897C>T alters the splice site in a manner that could affect the protein sequence and could be disease causing. The change is located between exons 4 and 5 and is likely to result in LGI1 mRNA truncation following exon 4, resulting in a mutant LGI1 protein truncated at amino acid 145 in the third LRR domain. Due to the unavailability of patient's cells that carry this intronic splice variant, we could not directly examine the mRNA sequence resulting from these splice site mutations. The screening of *RTN4R* and *LGI1* genes did not indicate that these genes are common variants associated with schizophrenia. However, the identification of the previously reported R399W mutation in *RTN4R* and the existence of three distinct LGI1 mutations raise the possibility that these genes may be involved in schizophrenia pathogenesis. Notably, this is the first time variants in LGI1 have been observed in a schizophrenic population.

To further explore the possibility that variants in *RTN4R* and *LGI1* may be disease causing, we performed a RVIS evaluation shown in Table 3. This genome‐wide scoring system assesses the functional variation in human genes based on the single nucleotide variants in the EVS. The RVIS percentile gives an indication as to whether a gene is “tolerant” or not to the presence of genetic variations. Lower scores indicate mutations are more likely to be disease causing. This score is significantly correlated with genes known to cause Mendelian diseases (Petrovski et al. 2013). The RVIS percentile for RTN4R is 7.05 and for LGI1 is 14.4; these are ranking scores and indicate that these two genes are not tolerant to changes and therefore may in fact be disease causing.

### LGI1 has reduced binding to mutant forms of NgR1 present in humans with schizophrenia

**Figure 2:**
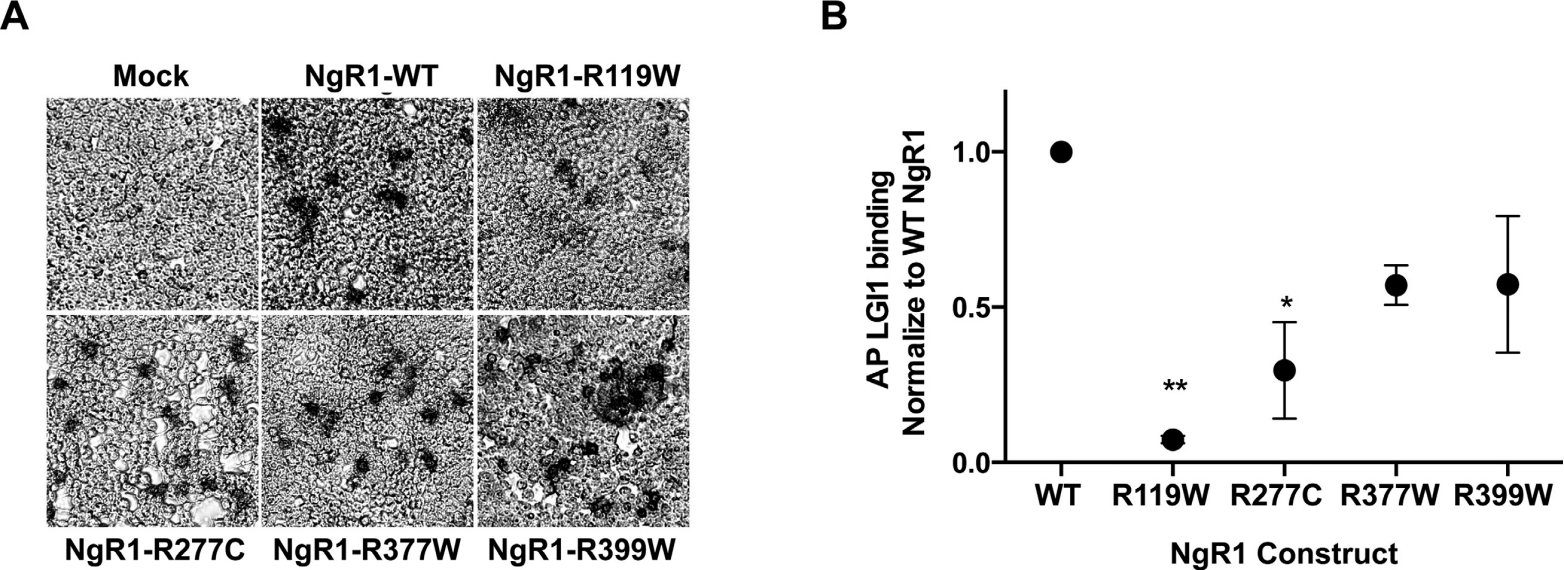
LGI1 has reduced binding to mutant forms of NgR1 found in humans with schizophrenia. (A) Sample images of COS7 cells incubated with 5 nmol/L AP‐LGI1 and stained with NCB‐BCIP to indicate LGI1 binding. Cells transfected with the indicated NgR1 constructs wild type or mutant. (B) Quantitative AP‐LGI1 binding to 293T cells expressing the indicated NgR1 WT or mutant constructs. Data represent the average value of 8 replicates in two experiments analyzed by one‐way ANOVA. **P < 0.01 and *P < 0.05, error bars indicate standard error of the mean.

Eight rare coding variants have recently been identified in *RTN4R* in SCZ populations. Four of these rare coding variants are predicted to be disease causing (Budel et al. 2008), including the R399W mutation confirmed in this study. Nogo66, MAG, and OMPG are all ligands for NgR1 (Fournier et al. 2001; Domeniconi et al. 2002; Wang et al. 2002b). Budel et al. tested the ability of these ligands to bind NgR1 mutants R196H, R119W, R377Q, R377W, and R399W, indicated in Figure 1 A. The authors noted impaired binding of MAG and OMGP to only R119W, whereas other mutations had normal binding. We previously identified LGI1 as an antagonistic ligand for NgR1 (Thomas et al. 2010), and here tested the ability of alkaline phosphatase tagged to LGI1 (AP‐LGI1) to bind to the NgR1 mutants with amino acid substitutions R119W, R277C, R377W, and R399W. Figure 2 shows that AP‐LGI1 binding to COS7 cells expressing mutant NgR1^R119W^ was dramatically reduced compared to AP‐LGI1 binding to wild‐type NgR1. AP‐LGI1 binding to NgR1^R277C^ is slightly reduced compared to wild‐type NgR1, but LGI1 binding to NgR1 mutants R377W and R399W was not significantly reduced.

### Two mutant forms of NgR1 associated with schizophrenia show functional impairment

**Figure 3:**
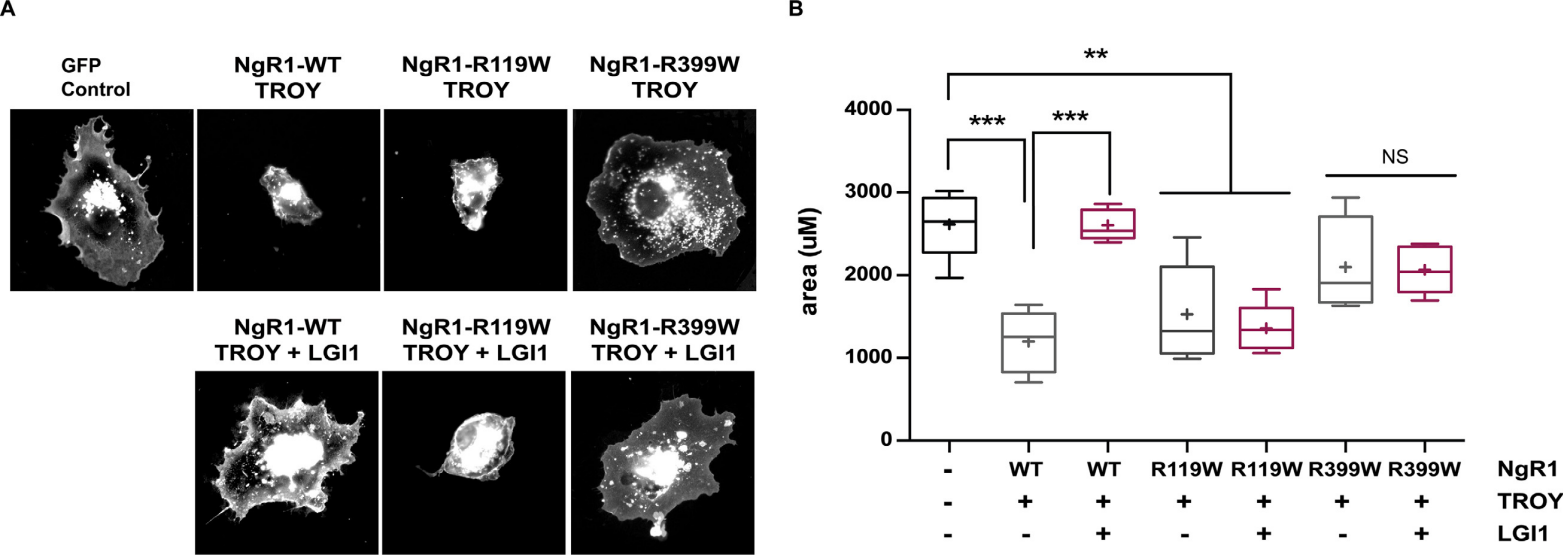
Impaired function of NgR1 mutants associated with schizophrenia (SCZ) in COS7 cell‐spreading assay. (A) Sample images of COS7 cells with cell surface labeled with rhodamine‐tagged WGA. Transfected constructs are indicated. (B) Quantification cell surface area. Values are the average area of >100 cells in five separate experiments. Analyzed by one‐way ANOVA with Bonferroni post hoc tests. ***P < 0.001 and **P < 0.01. Significant differences are indicated on the graph. NS = not significant. There are no differences in cell size between GFP control cells, NgR1^R399W^ + TROY, and NgR1^R399W^ + TROY + LGI1.

NgR1 and TROY function as coreceptors that activate RhoA, thereby altering the actin cytoskeleton (He and Koprivica 2004). COS7 cell spreading, assessed by measuring surface area occupied by cells, can be used as a surrogate for RhoA activation (Zeinieh et al. 2014). Expression of NgR1 together with TROY significantly reduces the surface area occupied by COS7 cells and additional expression of LGI1 recovers cell area to the control cell size. We tested the ability of the mutant NgR1^R119W^, which does not bind LGI1 and the mutant NgR1^R399W^, which does bind LGI1, for their ability to alter cell spreading, in the presence of TROY and in the absence and presence of LGI1. Figure 3 shows that in the absence of LGI1, wild‐type NgR1 and NgR1^R119W^ show significantly decreased cell spreading, whereas NgR1^R399W^ had no effect on cell size. This suggests that NgR1^R119W^ is capable of functionally interacting with TROY and activating RhoA, whereas NgR1^R399W^ is defective in this property. When expressed with LGI1, the cell contraction induced by wild‐type NgR1 was relieved, whereas that decrease in cell spreading induced by NgR1^R119W^ was not. These findings are consistent with the defect in LGI1 binding to NgR1^R119W^ noted above. Coexpression of LGI1 with NgR1^R399W^ had no effect on cell size, suggesting that a downstream signaling mechanism is impaired in this mutant. Taken together, these data suggest that variants in NgR1 alter LGI1 binding and RhoA signaling properties in distinct ways.

## Discussion

We performed two separate screens of SCZ populations for deleterious variants in *RTN4R* (which encodes NgR1) and *LGI1* (encoding LGI1), an antagonistic ligand for NgR1. Within the COS population, we identified one rare coding variant, c.1195C>T in *RTN4R*, that results in amino acid substitution R399W (Fig. 1A and Table 2). The same variant was previously identified in a schizophrenic patient and predicted to be harmful (Budel et al. 2008). Confirming this rare variant in a second patient adds confidence to hypothesis that NgR1^R399W^ contributes to disease progression. While it is noteworthy that the unaffected parent of this patient also carries the c.1195C>T variant in *RTN4R*, schizophrenia is a multifactorial disease with both genetic and environmental conditions, and the lack of symptoms in the father does not rule out the role of NgR1 and this variants in disease progression. We tested the function of the NgR1^R399W^ mutant proteins in two separate assays and found that this mutant had impaired RhoA activation properties (Fig. 3). The mutation is located in the stalk domain of NgR1 responsible for binding coreceptors p75NTR and TROY. We tested the ability of NgR1^R399W^ to bind the ligand LGI1 and found no impairment. Interestingly, we tested the ability of NgR1^R399W^ to decrease COS7 cell size, when coexpressed with TROY we found no change in cells size compared to control cells, indicating NgR1^R399W^ cannot mediate RhoA activation, possibly due to an inability to bind to TROY.

In addition to testing the rare coding variants in NgR1 that we identified in our patient cohort, we also tested three other NgR1 rare coding variants that had previously been identified in SCZ populations. The R119W and R277C mutations are in the ligand‐binding domain of NgR1 and R377W and R399W are located in the stalk domain (Fig. 1A). The mutants NgR1^R119W^ and NgR1^R277C^ showed a significant deficit in LGI1 binding, whereas binding to NgR1^R377W^ and NgR1^R399W^ were not altered (Fig. 2). The NgR1^R277C^ mutant produces an unbound cysteine that may cause aberrant disulfides and thereby produce major structural changes, beyond what would be expected from simple amino acid deletion or substitution. Budel et al. reported that binding of MAG and OMGP to the NgR1^R119W^ mutant is dramatically reduced but that Nogo66 binding was normal (2008). We previously reported that LGI1 and Nogo66 compete for binding to NgR1 and here report that NgR1^R119W^ is defective in binding to LGI1. Taken together, these data suggest that distinct portions of the LGI1 molecule share binding sites with MAG and OMGP versus Nogo66.

A functional link between SCZ and NgR1 is supported by the detection of rare variants in humans patients both here and in other work (Sinibaldi et al. 2004; Hsu et al. 2007; Budel et al. 2008). Additionally, we find that variants in *RTN4R* are likely to be disease causing. Furthermore, postmortem expression of NgR1 mRNA is reduced in SCZ patients compared to controls (Fernandez‐Enright et al. 2014). In this study, we also reported the first instance of genetic variants in *LGI1* in SCZ patients. We uncovered two coding variants in *LGI1* and one intronic variant in *LGI1*. In human epileptic patients, 40 variants affecting 36 different sites have been identified. Schizophrenia and epilepsy are both developmental disorders of the central nervous system caused in part by improper circuit formation and impaired synaptic transmission. The two disease states can also be comorbid, and the prevalence of SCZ in patients with temporal lobe epilepsy is 7% (Clancy et al. 2014). To date, no epileptic patients expressing mutant LGI1 have been reported with a SCZ diagnosis. However, almost all LGI1‐ADLTLE patients experience auditory or other sensory hallucinations preceding seizure onset. Additionally, several other comorbidities in have been described in ADLTLE patients and their families with LGI1 variants. A set of LGI1‐ADLTLE patients have delayed language‐processing responses in fMRI (Ottman et al. 2008) and impaired language processing can be a SCZ symptom. In a separate study LGI1‐ADLTLE patients scored lower on measures of auditory processing than controls (Pisano et al. 2005). In one Japanese family, nine family members with variants in LGI1 had psychotic symptoms including emotional outrage and explosive violent behaviors, five of these nine patients also have epilepsy (Kawamata et al. 2010). In a Dutch family, half the ADLTLE patients with variants in LGI1 also suffer from attention deficit disorder (Berghuis et al. 2013). Patients with LGI1 variants causing ADLTLE also suffered from migraines (de Bellescize et al. 2009).

The connection between epilepsy and SCZ also goes in the other direction. A case study of a patient with a 22q11 deletion, the chromosomal position of *RTN4R* shows a specific association between psychosis and epilepsy (Tastuzawa et al. 2015). Patients with the 22q11 deletion subtype of schizophrenia respond normally to the antipsychotic clozapine, but have an increased risk of seizures as a treatment side effect (Butcher et al. 2015).

LGI1 and NgR1 both contribute to the development and maintenance of normal synapses (Raiker et al. 2010; Wills et al. 2012; Lovero et al. 2015). NgR1 deletion in mice results in changes in plasticity, memory formation, and social behavior (McGee et al. 2005; Karlén et al. 2009; Lazar et al. 2011). Some LGI1 variants lead to ADLTLE and antibodies directed against LGI1 in human limbic encephalitis lead to psychosis and seizures, indicating a role for LGI1 synaptic in connectivity (Kegel et al. 2013; Deakin et al. 2014). Overall, our findings indicate that NgR1 and LGI1 contribute to appropriate CNS circuitry formation and maintenance and perturbing either protein function in this pathway can contribute to different disease states. Identification of rare coding variants found in disease provides a useful tool for understanding in vivo protein function in disease progression.

## Conflict of Interest

None declared.
